# Repetitive Traumatic Discopathy in the Modern-Era Tennis Player

**DOI:** 10.7759/cureus.9783

**Published:** 2020-08-16

**Authors:** Brian Fiani, Ryan Jarrah, Amelia Wong, Adam Alamah, Juliana Runnels

**Affiliations:** 1 Neurosurgery, Desert Regional Medical Center, Palm Springs, USA; 2 Literature, Arts, and Sciences, University of Michigan-Flint, Flint, USA; 3 Osteopathic Medicine, Western University of Health Sciences, Pomona, USA; 4 Kinesiology, Michigan State University, East Lansing, USA; 5 Medicine, University of New Mexico, Albuquerque, USA

**Keywords:** degenerative disc disease, disc disease, discopathy, athletics

## Abstract

Degenerative disc disease is more prevalent among athletes than the general population. Repetitive traumatic discopathy is a pattern of injury that has been described in athletes participating in sports that impart repetitive mechanical forces on the lumbar spine. Hence, tennis players may be particularly susceptible to repetitive traumatic discopathy due to the fast-paced nature of the modern tennis match. Recent biomechanical studies have identified the lumbar spine as the focal point of motion during tennis strokes, and the lumbar spine is notably the most frequent location of injury observed in tennis players. In this comprehensive review, we examine current evidence and discuss the epidemiology, pathophysiology, biomechanics, diagnosis, and treatment of repetitive traumatic discopathy in tennis players. Additionally, we outline considerations for rehabilitation and return to the tennis court after operative management.

## Introduction and background

While athletic activity has been well characterized for its physiological benefits in spinal health, overuse has led to spinal degradation in much of the athletic population. Repetitive traumatic discopathy (RTD) describes degeneration or herniation of the intervertebral discs following repeated traumatic movements that lead to pain, instability, and deterioration of the spine. The clinical manifestations of RTD result from the cumulative effects of repetitive overload and stress affecting the spine [1]. RTD occurs most frequently at the level of the spine that is subjected to the most substantial mechanical stress. The volume and stability of the intervertebral disc tissue decreases with these repetitive traumatic external movements. Due to the dramatic physical nature of the sport, RTD and disc degeneration are significantly more prevalent in elite athletes (75%) than in non-athletes (31%), leading to high proportions of back pain among this population [2]. Various studies have shown that up to 75% of athletes have had one or more episodes of back pain, mainly due to the physical demands placed on the spines of athletes as they train and compete. In fact, in one study among active athletes, back pain was as high as 85%, suggesting a correlation to athletic activity [3].

The prevalence of lumbar degenerative disorders has been well characterized among athletes across all sports. One noteworthy study during the 2000 Olympic Games in Sydney analyzed the lumbar spines of athletes two weeks before and after the start of the competition. The results found that 36% of athletes demonstrated severe disc degeneration while 26% showed mild disc degeneration. When examining disc height reduction, 68% of athletes showed symptoms at a single level, while only 19% of athletes had normal disc heights at all levels. When examining disc displacement, 58% of athletes showed some degree of disc displacement, most of which were disc bulges [4]. The results of these studies are well-supported by current literature, suggesting RTD is more prevalent in athletes than the general population [2]. 

These findings are very relevant to specifically the sport of tennis, due to the unique biomechanical physical demands involved in serving and groundstrokes. The back, trunk, and hips are essential in tennis because they serve as the center of rotation, and transmit the forces generated in the lower extremities to the shoulders and arms. One of the most common areas of injury in the central region of tennis players is the posterior midline paraspinal musculature. This muscle group is used for overhand serves, charging the net, and dropping straight back for a volley arms [5]. One study analyzed the degree of lumbar spine injury in asymptomatic adolescent tennis players and found that of the 33 players examined, almost all showed several RTD-like symptoms or diagnoses. Nine players showed pars lesions, predominately at the L5 level, while 23 participants showed symptoms of early facet arthropathy occurring at L5/S1. Early facet arthropathy was further classified as mild degeneration (20/29), moderate degeneration (9/29), sclerosis (20/29), and hypertrophy of the facet joint (24/29). According to these results, RTD abnormalities were common in the tennis players, especially in the lower lumbar spine area and almost exclusively at L4/5 and L5/S1 levels [2].

RTD’s potential in shortening the careers of tennis-players while increasing susceptibility to long-term deterioration of the spine, makes its recognition essential to players and practitioners alike. With increasing studies showing that RTD can allow for secondary pathologies, such as facet joint arthropathy and synovial cysts, the importance of a clinical understanding of RTD is essential for the longevity of athletic careers. Our literature review will analyze spinal degradation secondary to tennis in order to provide medical experts and athletes with knowledge of the pathophysiology, biomechanics, and treatment of RTD. Herein, we will characterize RTD in the context of tennis through a comprehensive and contemporary review of the available literature to date.

## Review

Pathophysiology

The lumbar spine is essential for generating force, transmitting force, and absorbing shock in bodily movements. Athletes across diverse sporting activities particularly subject their spines to repetitive demands, thereby increasing musculoskeletal stress and risk of RTD compared to controls [2,6]. Sports that have demonstrated this pathology include tennis, baseball, combat sports, golf, weight lifting, gymnastics, skiing, and volleyball [4,7-11]. Despite the many cited health benefits of tennis, players are also exposed to substantial spinal loading and torsional stress due to the compounding effects of dynamic truncal motion and the requisite magnitude of force necessary to serve and hit a tennis ball.

The intervertebral disc (IVD) is comprised of three anatomic components: a gelatinous nucleus pulposus, surrounding concentric lamellar fibers of the annulus fibrosus, and cartilaginous endplates that flank the disc cranially and caudally to anchor it to the vertebral bodies [12]. A healthy nucleus pulposus is rich in collagen type II and negatively charged proteoglycans, of which aggrecan is the most abundant type [13]. These proteoglycans help stabilize the IVD during spinal loading by creating osmotic pressures of 420-450 mOsm to bind water in the extracellular matrix and produce intradiscal hydrostatic pressures [14]. This intradiscal pressure is then distributed vertically and laterally to the endplates and annulus fibrosis, respectively, to allow for spinal flexibility, shock absorption, and stability [15]. Cleavage of aggrecan from its hyaluronic acid backbone in degenerating discs impairs its ability to aggregate and bind water, thus rendering it ineffective at generating and distributing pressures within the IVD [16]. This functional impairment is further compounded by a reduction in proteoglycan synthesis, which normally reacts to increased hydrostatic pressures by stimulating an anabolic response [17]. Although degenerating discs retain the ability to synthesize new matrix, the production of collagen is modified from type II to the more fibrous type I and proteoglycans are altered from aggrecan to versican, biglycan, and decorin. The resulting dehydrated, increasingly fibrocartilaginous IVD is less stable and triggers a harmful positive feedback mechanism that further promotes intervertebral disc disease (IDD) [15,18].

Mechanical overloading, the subsequent release of matrix hydrolysis products, and progressive expression of inflammatory cytokines (e.g. IL-1, IL-6, TNF-α), and induced nitric oxide synthase, have been identified as inciting factors for RTD [12,18,19]. The pathogenesis involves the upregulation of matrix-degrading enzymes Matrix Metalloproteinases (MMP) and A Disintegrin And Metalloproteinases with Thrombospondin Motifs (ADAMTS) with an insufficient parallel increase of Tissue Inhibitor of Metalloprotein (TIMP) enzymes, particularly within the nucleus pulposus and inner annular layers [13,20]. Increased expression of MMP-1, -2, -3, -7, -8, -9, -10, -12, -13, and -14 has been observed but there is currently no consensus as to which MMP(s) are the principal actors during IVD matrix degeneration [20]. ADAMTS-4 and -5 are classified as the primary aggrecanases due to their high efficiency of cleaving aggrecan. While increases in ADAMTS-4 have been widely observed in degenerate discs, similar rises in ADAMTS-5 have not been seen, and its role in RTD requires further investigation [20,21]. Rises in TIMPS-1 and -2, potent inhibitors of MPPs but not aggrecanases, are seen with increasing RTD severity. Interestingly, expression of the potent aggrecanase inhibitor TIMP-3 was not observed, suggesting that ADAMTS-4 may play a pivotal role in IVD matrix degeneration [13].

Spine biomechanics in tennis

The game of tennis also has a clear biomechanical component that could contribute to a sports-related injury, particularly with RTD. With no time limit to matches, the game is unpredictable and can have a huge physiological and biomechanical demand. According to modern tennis instruction, a player’s success is greatly influenced by the ability to use correct body mechanics to maximize precision, accuracy, and power in stroke/swing production [22]. Modern tennis today requires several components, such as speed, strength, and flexibility, each of which presents biomechanical properties [23]. Speed in tennis refers to not only the speed in maneuvering along the court, but the ability to adjust the feet, knees, arms, and the spine to produce and counter shots. Modern instruction requires mastery of quick linear movements along with lateral and multidirectional motions that could leave the spine vulnerable for injury such as RTD [23].

Furthermore, a typical five-second point may require as many as four changes in body orientations, making speed/agility critical to a player’s success while also making the body susceptible to possible strain and injury. Flexibility in tennis refers to a player’s ability to stretch and extend their extremities to reach and return shots. A player’s flexibility in tennis may lead to joints and muscles reaching their full range of motion (ROM), a physical demand that could lead to RTD and other musculoskeletal maladaptations [23]. While tennis players typically have a larger ROM in their internal shoulder movement compared to other athletes, they have a smaller ROM in their hamstrings compared to these same athletes [23]. This reduced ROM is related to the fact that a low starting position is required for explosive movement, yet this also means that the hamstrings are in a shortened and more contracted state for more extended periods. This biomechanical aspect could eventually lead to musculoskeletal issues such as RTD [23]. Modern tennis instruction also requires strength for performance enhancement. Tennis requires repetitive movements of the body that requires the strength to initiate powerful swings. Instructors will demonstrate that a firm grip on the racket is fundamental for a player’s performance and success [23]. At the elite level, this grip strength has even been estimated at over 600N of force [23]. Top male players can serve the ball as fast as 130-140mph [23]. The strength of a player determines the physical demand they place on their body to produce high-velocity shots, along with how they generate power within their bodies. For example, players in weaker physical condition may need to rotate or adjust their body more dramatically to counter the shots of stronger players. Consequently, upper and lower body strength are major limiting factors in tennis performance and key parts to instructional fitness.

There are five main types of swings/strokes in the game of tennis that involve different biomechanical adjustments: forehand strokes, backhand strokes, serves, volleys, and overhand smashes [24]. Each of these swings requires a different repetitive stance and modification of the body position, making the spine profoundly vulnerable to overuse and eventual injuries such as RTD. A forehand and backhand fall under the umbrella term of a “groundstroke,” yet present different swing mechanics and kinematics. Both groundstrokes follow a kinetic chain of events where force is generated from the legs and then transferred to the hips, back, upper arms, and finally, the lower arm in quick succession [22]. A forehand is a swing with the dominant arm, where the ball is approaching the preferred side of the player and is in position to be struck. The forehand stroke involves either an open or closed stance of the body. An open stance is where the body is openly facing the ball in a direction where the ankles and hips are parallel to the net while a square stance is where the ankles and hips are faced more perpendicular to the net. During the forehand swing, the racket arm is extended back as the ball approaches the player, and as the player strikes the ball, the kinetics of the shoulder extends and rotates into a follow-through motion. This requires a twist in the lumbar spine to reciprocate the power of the ball and to create a power velocity that can be returned. Forehand swings impact the spine by causing an increase in upper lumbar extension and rightward axial rotation along with an increased lower lumbar right rotation/lateral flexion movements compared to a backhanded stroke [25].

A backhand groundstroke is a swing of the racket where the ball is across the direction of the dominant arm, requiring the back of the racket-hand to proceed with the palm. This stroke is performed with two hands holding the racket, increasing the grip force. The backhand requires further rotation of the spine, as the arms and body turn as a unit at almost a 90º angle from the starting position which may cause an increase in axial rotation forces during the initiation of the swing. The backhand also showed great upper lumbar leftward rotation while forehands show greater lumbar right lateral flexion forces [25]. This is possibly where many spinal discopathies originate from, as it requires an unnatural and eccentric swing of the body [25]. While forehand and backhand groundstrokes typically produce lower magnitudes of lumbar force than the serve, they were also found to be 1.62 times more prevalent during competitive matches and likely cumulatively induce injury [26].

Serves are what begin each tennis point, and they follow a step-wise process that results in eight phases and three stages: preparation, acceleration, and follow-through [27]. In phase 1 (start), the lumbar spine starts as the base of support and stability while the body is aligned to use the ground for force/power generation [27]. In phase 2 (release), the ball is released from the non-dominant hand as the muscle activation of the right erector spinae increases from the beginning to the end of the serve. Toes are located laterally to the overhead stance of the server to allow for proper arm abduction and subacromial humeral position [27]. In phase 3 (loading), potential energy is gathered with the knees being bent at and angle less than 15º, while the pelvis and shoulder are at a tilt that is lateral to the rear [27]. The spine then moves into hyperextension, along with ipsilateral lateral flexion movements, and ipsilateral rotations [27]. The torso and spine rotate slightly in a counter-motion as energy is being stored for contact of the ball. This plyometric stretch-shortening pattern has been characterized to cause spinal pathologies such as spondylosis and potentially RTD [27]. In stage 5 (cocking), the focus turns to the shoulders, as the racket arm is abducted at around a 110º angle at an external rotation angle of 172º. The wrist and the spine are extended to allow for greater vertical ground reaction forces. In phase 5 (acceleration), the server is just about to strike the ball, as he/she jerks, jumps, and extends for maximum velocity [27]. The biomechanics for this phase onward will be similar to that for the overhand smash. This phase depends on both strength and neuromuscular coordination along with the momentum, potential energy, and angular acceleration of the racket generated from the previous five phases. In this phase, high muscle activity is seen in the pectoralis major, subscapularis, latissimus dorsi, and serratus anterior as the vertical force production produced can be over double the server's body weight [27]. According to sports scientists, trunk muscles show their highest activation levels during this phase as the acceleration of the racket is accompanied by rapid lumbar spine rotation reversals followed by right lateral flexion or left lateral flexion movements [27]. This causes the trunk to be hyperextended, transferring torque onto segments of the spine in a way that could lead to RTD [27]. In phase 6 (contact), the player strikes the ball using the potential energy generated from the kinetic chain of events prior. The trunk is tilted at a 48º angle to the horizontal plane of the hips, while the racket-arm is abducted at a 110º for optimal contact [27]. Next, the server enters the most biomechanically violent stage: the follow-through. It is during the follow-through phase that the highest torsional and shearing forces are transmitted onto the vertebral bodies. In phase 7 (deceleration), the glenohumeral shoulder joints and forearm pronation continue to create a coupled motion known as a long-axis rotation. Sometimes up to ¾ of the server’s body weight is required to stabilize the scapular region during the deceleration phase after contact [27]. The right-internal oblique and right erector spinae become active in order to rebalance spinal posture, adding possible strain to the region [27]. As the server enters phase 8 (finish), larger eccentric forces become active. The landing foot lunges forward, requiring horizontal braking forces, while the trailing foot kicks backward, and the center of mass is pushed towards the front of the body. Over 300 Nm of deceleration forces are shared between the arms and the trunk, adding further areas in tennis for spinal injury such as RTD [27].

Volleys, unlike other strokes, require the player to approach the ball and don’t require big swing-like motions. This stroke is usually performed near the net and before the ball bounces. Despite being a swift movement, this stroke involves nine muscle groups, with the power being generated from the legs pushing the body forward along with the shoulders turning and the forearm extending [28]. The spine can also be impacted, particularly near the infraspinatus muscles at the shoulder. However, the rotation of the body is much less with this stroke, meaning less strain on the spinal region.

Diagnostic techniques

Electromyography (EMG) studies have helped elucidate the pathogenesis of RTD in tennis players by identifying the specific muscles and activation sequence necessary to stabilize the trunk while performing various tennis motions. Specifically, inadequate or improper activation of the erector spinae, rectus abdominus, and external obliques increases muscular stress and generates harmful spinal loads that are highlighted in EMG imaging [29]. As mentioned, during the serving motion (specifically stage 5), the trunk hyperextends, laterally flexes, rotates, and generates spinal loads of up to 3000 N [29,30]. This makes the service motion highly significant when using EMG analysis to study RTD. Furthermore, the rapid reversal of lumbar rotation and flexion during the follow-through phase allows torsional and shearing forces onto the vertebrae that are seen during EMG imaging. Noticing the simultaneous loading of the lumbar spine in multiple directions has previously been linked to a higher incidence of disc strain, vertebral failure, and subsequent lumbar disc pathology [31,32]. Decreased trunk activation, uncoordinated contraction patterns, and lack of extensor endurance have been associated with spinal instability and LBP by using EMG studies [29].

Magnetic resonance imaging (MRI) is widely accepted as an essential diagnostic tool for imaging spinal abnormalities. Intervertebral disc degeneration (IDD) is characterized by the progressive loss of MRI signal intensity in the disc and vertebral endplates [33,34]. This is consistent with studies that have shown that nuclear brightness on MRI correlates directly with proteoglycan content but not with water or collagen [34,35]. Disease severity is often described by the Pfirrmann system, which assigns grades I-V based on the visual evaluation of disc homogeneity, ability to distinguish the nucleus and annulus, and magnitude of disc height reduction on T2-weighted MRI [34]. Bone marrow changes in the vertebral endplates often accompany IVD degeneration and are classified into Modic Type I-III. Variations of signal intensities of T1-weighted and T2-weighted MRI represent progressive IDD resulting from increased subchondral bone perfusion due to microinflammation, bone marrow fatty degeneration, and subchondral bone hardening [18,33]. Advances in MRI T2 mapping and MRI T1ρ mapping eliminate the subjective element of visual assessment as mapping involves digitizing matrix components to quantitively evaluate the metabolite concentrations within IVD tissues and degree of cartilaginous degradation, respectively [18].

Radiographic changes in the lumbar spines of athletes are well documented. Although IVD degeneration is also a consequence of normal aging, the prevalence of disc degeneration is more common in athletes than non-athletes [9,36]. Comparison of T2-weighted MRI lumbar spines in asymptomatic adolescent elite tennis players demonstrated a higher prevalence of disc degeneration than asymptomatic non-elite athletes (62.2% vs. 37%) [37]. Degenerated discs were predominantly found at the L5/S1 level and exhibited mild degrees of degeneration [37,38]. Other sports such as gymnastics and cricket showed similar prevalence and patterns of disc degeneration and suggest that axial loading in these sports may be similar to tennis [37]. It is important to note, however, that the presence of degenerative radiographic change is also prevalent in asymptomatic individuals and thus does not correlate with clinical symptoms of low back pain (LBP) [39]. Contrast-enhanced imaging such as discography can be used to evaluate IVD-associated LBP. Tears in the IVD are detected as instantaneous dispersion of contrast medium as opposed to gradually in healthy discs. Additionally, the formation of granulation tissue with neovascularization to repair these radiating tears appears as bands on contrast-enhanced MRI and is useful in diagnosing IDD [18]. However, studies rely on subjective reproduction of pain and are associated with varying false-positive rates of 10% to 80% depending on a patient’s history of low back pathology [18].

Surgical intervention

When back pain has lasted over six months, and rehabilitation medicine, oral regimens, and injection administrations fail to provide relief, then surgical intervention may be required to eliminate pain-like symptoms and spinal limitations of RTD, or if there is a neurological deficit secondary to RTD. As previously mentioned, the repetitive motion of tennis swings can cause RTD through hyperextension and eccentric rotations of the spine. RTD can formulate into disc herniations, causing discs between vertebrae to displace from their normal location and increase pressure on spinal nerves causing radiculopathy. Radiculopathy from RTD can cause pain, neurological sequelae, and symptoms of numbness, tingling, or weakness that radiate in specific dermatomes and myotomes. Such injury can limit the player’s spinal rotations and inevitably disrupt their athletic performance. Failure to resolve RTD could result in progressive worsening back pain or radiation of symptoms. There are two main minimally invasive surgical techniques to resolve RTD and other spinal discopathies: a discectomy and fusion or an artificial disc replacement [40].

Discectomy and fusions are surgical procedures that involve resection of the damaged intervertebral disc, placement of an interbody device, and forming bony vertebral fusions with a mechanical construct that causes ossification and arthrodesis to add structural support to the spine. Bone graft may be used to help create the permanent fusion. A potential consequence of surgery may be that a player’s physical range of motion and lateral spinal rotations may be compromised due to the increased stability of the spine. Such rigidity could tremendously affect their optimal performance, but the goal is to give the patient the ability to play without the burden of spinal pain.

Artificial disc replacement or total disc replacement (TDR) is the alternative surgical technique that may provide continued range of motion at that spinal level, and thus, continued rotational force for tennis swings. TDR became FDA-approved in 2005 and has been implemented in sports medicine clinical trials for over 15 years with great success [41]. The injured/displaced disc is resected and replaced with an artificial device that mimics the anatomical structure of natural spinal discs. Tennis players strongly prefer this technique because it could result in restoring normal spinal motion and rotation [42]. Also, recovery times tend to be quicker than fusion surgeries. Moreover, with a fusion, there is the probability that the vertebrae above or below the fused section have an added physical demand and degeneration, referred to as adjacent level disease, especially if there is violation of adjacent supporting ligaments [42].

Tennis rehabilitation and use of external orthoses

Physical rehabilitation is a necessary process that serves to ensure a safe return to active participation in tennis. The rehabilitation process for RTD varies depending on the needs of the patient. Many factors should be considered for a tennis player engaging in RTD rehabilitation, such as the athlete’s physical development (if still of younger age), chronicity of the injury, the proper technique and mechanics, associated injuries, and appropriate equipment. The three phases of rehabilitation include an acute phase, a recovery phase, and a maintenance phase. The acute phase involves the initial treatment, which is conducted to simply reduce the symptoms of injury as well as, control tissue injury. The recovery phase consists of the process of tissue healing, which includes reducing tissue overload. The maintenance phase involves directing one’s efforts from therapeutic activities towards the progression of sport-specific gains of function. The completion of the entire rehabilitation process leads to the return of play [3].

Mild to severe RTD may start with associated lumbar muscular strain, which is the most common back injury in tennis players. Rehabilitation for this injury should include relative rest, pain relief, ice application to relieve temporary muscle spasms, etc. Once pain is relieved, gradual flexibility followed by a strengthening regimen should occur, specifically with an emphasis on strengthening lower limbs and shoulder to avoid a weak link in the kinetic chain that could potentially place more strain on lower back muscles [43]. Tennis players who suffer from lumbar disc degeneration injury, which is most caused by the service motion, may experience back pain, leg pain, or a combination of both. Rehabilitation includes rest and pain control, which may consist of anti-inflammatory medications or the sparing use of opioid medications for players with severe pain [44]. Physical therapy should consist of proper trunk and abdominal flexibility, and core exercises to unload the lumbar disc. Diaphragm training should be incorporated into the therapy process. The athlete should also correct his or her biomechanics and tennis technique to ensure that a similar injury will be less likely to occur [3].

One vital part of the rehabilitation process in spine-related injuries is the use of external orthoses, such as lumbar back braces. Back bracing can assist in injury prevention by providing support to muscles in the back region. No literature to date specifically examines the efficacy of external orthosis usage in tennis players for pain management, decreasing opioid usage, preventing disc degeneration from RTD, or its impact on the return to play rates. Additional studies are needed to find if those benefits are achieved. However, the general principles of usage would apply. External orthoses may apply pressure on lumbar muscles, preventing them from making any sudden, painful movements. Although a brace limits sudden movements, which may lead to injury, for the high performing tennis player it should provide enough flexibility for one to freely engage in the proper ranges of motion. An external orthosis may help players to maintain proper form and posture. RTD rehabilitation is a robust process that requires pain control, physical therapy, optimal tennis technique, and proper use of external orthoses to ensure the safest and most efficient recovery possible [45].

## Conclusions

Though genetics and age are strong risk factors for degenerative disc disease, athletes are more susceptible to accelerated lumbar degeneration than non-athletes. RTD is a pattern of injury first described in golfers after chronic, repetitive spinal loading, and torsional stress. In a similar manner, tennis players subject the lumbar spine, specifically the IVD, to cumulative trauma producing early degenerative intervertebral disc disease. The IVD is composed of proteoglycans that provide stabilization during spinal loading by creating high osmotic pressures that produce strong intradiscal hydrostatic pressure forces. However, during mechanical overloading, the release of matrix hydrolysis products, expression of inflammatory cytokines, and induction of nitric oxide synthase have been identified as inciting factors for RTD. In this review, we have discussed the spine biomechanics that specifically play a role in the development of RTD.

Clinicians can help tennis players mitigate lumbar degeneration by identifying early risk factors and patterns of injury. More studies are needed for the future consideration of how to help athletes before their pathogenesis of IVD progresses to the point of surgical intervention. Prevention should be the key focus of clinicians moving forth. Perhaps early introduction of physical therapy techniques for core exercises and diaphragm training, along with the early introduction of external orthoses could mitigate IVD injury. New minimally invasive techniques, such as intradiscal cellular injections, could be the future treatment method of choice if additional studies show supporting evidence. Further studies investigating the outcomes from intradiscal cellular injection techniques for treating this pattern of injury are necessary to guide management and to see how a novel technique such as this might supplement current treatment practices.
